# Correlation between the HIF-1α/Notch signaling pathway and Modic changes in nucleus pulposus cells isolated from patients with low back pain

**DOI:** 10.1186/s12891-020-03505-w

**Published:** 2020-07-28

**Authors:** Zekang Xiong, Jun Ding, Jinge Zhou, Sheng Yao, Jin Zheng, Xiaodong Guo

**Affiliations:** 1 Department of Orthopedics, Union Hospital, Tongji Medical College, Huazhong University of Science and Technology, 1277 Jiefang Avenue, Wuhan, 430022 China; 2 Department of Neurology, Wuhan No.1 Hospital, 215 Zhongshan Avenue, Wuhan, China; 3 Department of Neurology, Union Hospital, Tongji Medical College, Huazhong University of Science and Technology, 1277 Jiefang Avenue, Wuhan, 430022 China

**Keywords:** Modic changes, HIF-1α, Notch signaling pathway, Nucleus pulposus, Endplate degeneration

## Abstract

**Background:**

The HIF-1α/Notch signaling pathway regulates cell proliferation, apoptosis, and metabolism in the intervertebral discs (IVDs) and is implicated in disc degeneration. The nucleus pulposus (NP) is an important structure adjacent to the IVDs. However, the role of the HIF-1α/Notch signaling pathway in NP cells obtained from patients with different Modic changes (MCs) remains unclear. The purpose of the present study was to investigate the role of HIF-1α and components of the Notch pathway in the NP obtained from patients with various MCs.

**Methods:**

A total of 85 NP tissue samples were collected from patients undergoing diskectomy for the treatment of low back pain. The NP tissues were divided into four groups based on the adjacent endplate degeneration, namely, MC I, II, III, and negative MC groups. The expression of HIF-1α and Notch-related components was measured and compared.

**Results:**

The expression of HIF-1α, Notch1, and Notch2 was gradually increased in the MC I and MC II groups compared with that in the negative MC group. HIF-1α and Notch-related components were rarely detected in the MC III group.

**Conclusions:**

The expression of HIF-1α/Notch increased in the NP cells of patients with MC I and MC II. HIF-1α and Notch-related components are potential biomarkers and the HIF-1α/Notch signaling pathway may serve as a promising therapeutic target for disc degeneration in patients with MCs.

## Background

Modic changes (MCs) are changes in the vertebral body marrow and endplate lesions that appear as visual signals on magnetic resonance imaging (MRI). MCs were first described and classified into 3 general types by Modic et al. in 1988 [1, 2]. Different Modic types may represent different stages of the same pathological process. Modic type 1 changes (MC I) are associated with fissuring of the cartilaginous endplate corresponding to endplate edema. Modic type 2 changes (MC II) reflect fatty replacement of the adjacent marrow. Modic type 3 lesions (MC III) are observed in vertebral bodies with sclerotic changes.

Previous studies investigating MCs have focused mainly on the imaging changes and the association with low back pain (LBP) [3, 4]. Repetitive loading and periodic injury resulting in inflammation are two major pathological factors that contribute to MCs [5, 6], and result in endplate changes and intervertebral disc (IVD) degeneration. The potential association between IVD degeneration and MCs requires further investigation. Therefore, the aim of the present study was to clarify the correlation between IVD degeneration and MCs, specifically by focusing on the changes in hypoxic biomarkers.

Hypoxia-inducible factor (HIF) is a master transcription factor that mediates the activation of coordinated cellular responses in order to adapt to hypoxic environments. The HIF proteins are heterodimers that consist of a regulable α-subunit and a conserved β-subunit, and can be subdivided into three types: HIF-1, HIF-2, and HIF-3 [7, 8]. The hypoxia response elements (HREs) are the final point of integration of signaling pathways regulated by oxygen [8, 9]. Briefly, α-subunits dimerize with β-subunits to bind to HREs, which activate the transcription of effector genes under hypoxic conditions. The rapid degeneration of α-subunits inhibits the transcriptional activity of HIFs under normoxic conditions [8, 9]. In nucleus pulposus (NP) cells, HIF-1 plays an important role in the regulation of biological behaviors, such as energy metabolism, matrix metabolism, radical dismutation, cell proliferation and survival [7, 10]. Risbud et al. [11] revealed that HIF-1α was stably expressed in human, rat, and sheep NP cells under hypoxic conditions, suggesting that HIF-1α may serve as a potential target for the prevention and treatment of IVD degeneration. Therefore, in the present study, HIF-1α was used as an index of ischemia and anoxia in NP cells in the IVDs.

Notch is a hypoxia-sensitive receptor protein that regulates the proliferation of progenitor cells. There are four types of Notch receptors, namely, Notch1, Notch2, Notch3, and Notch4. Several studies have investigated the relationship between HIF-1 and Notch in physiological and pathological conditions [12–14]. Hypoxia activates the Notch signaling pathway to maintain IVD cell proliferation and accelerates catabolism. In the NP and annulus fibrosus (AF) in rat disc tissue, the hypoxia-induced increase in Notch mRNA expression can be blocked by a Notch signaling inhibitor. Furthermore, the expression of the Notch target gene HES1 was induced by hypoxia. Moreover, inhibition of Notch signaling inhibited the proliferation of disc cells. Analysis of human degenerated disc tissue revealed that the expression of Notch signaling proteins was increased. Additionally, the increased expression of inflammatory cytokines promoted Notch signaling in degenerated discs [15]. Therefore, the HIF-1α/Notch signaling pathway plays an important role in disc degeneration, and may serve as a potential therapeutic target for the restoration of cell numbers in degenerative disc disease.

The present study investigated the expression of HIF-1α and Notch in the bulging discs adjacent to end plates with MCs. Furthermore, we explored the relationship between MCs and disc degeneration using imaging, biochemical, and immunohistochemical methods to determine whether the expression of HIF-1α and Notch may aid the diagnosis and treatment of degenerative disc diseases.

## Methods

### Human tissue collection

The Ethics Committee of Huazhong University of Science and Technology approved this study and waived the requirement for informed consent. A total of 85 surgical NP tissue samples were collected from patients undergoing diskectomy for the treatment of LBP between January 2013 and January 2016. Each sample was obtained from the protrusive region of the IVD. The average LBP intensity was documented on a 0–10 numerical rating scale (NRS) as follows: 0 = no pain and 10 = worst possible pain (Table 1).

**Table 1:** Characteristics of the 85 patients included in the study

Modic type	Sex	n	Age	Sample level	Population	NRS score
Control Group	M F	5 15	48.81 ± 9.93	L1/2	2	3.3 ± 2.1
				L2/3	1	
				L3/4	3	
				L4/5	8	
				L5/S1	6	
Modic Type 1	M F	9 6	42.6 ± 12.88	L1/2	2	4.2 ± 1.6
				L4/5	9	
				L5/S1	4	
Modic Type 2	M F	10 30	55.69 ± 11.03	L1/2	2	3.6 ± 1.7
				L2/3	4	
				L3/4	5	
				L4/5	12	
				L5/S1	17	
Modic Type 3	M F	4 6	64.6 ± 7.3	L2/3	1	4.3 ± 1.7
				L4/5	6	
				L5/S1	3	

Values are presented as the mean ± SD

**Fig. 1:**
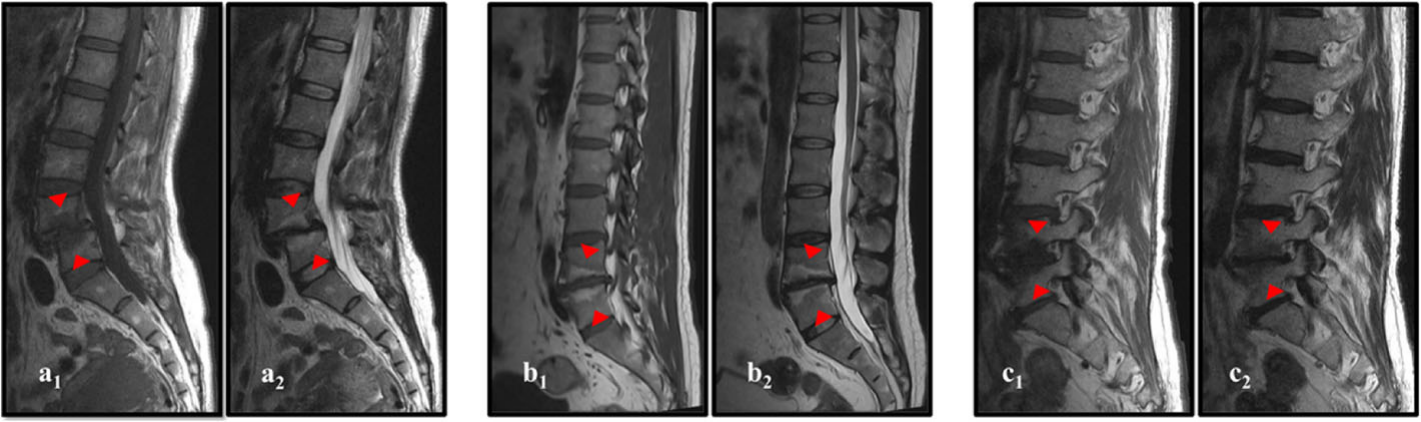
Representative T1- and corresponding T2-weighted images of different MCs. The red arrows indicate the positions of the MCs. MCs, Modic changes

As shown in Fig. 1, patients with MC I, MC II, and MC III were included in the present study according to the inclusion criteria for MCs on MRI. The exclusion criteria were as follows: mixed MCs, ankylosing spondylitis, scoliosis, vertebral fractures, lumbar spine infection, spinal tumors, metastatic lesions, and other spine-related diseases; diabetes, hypertension, and other relevant medical history; history of spinal surgery, smoking, alcoholism, or drug use; psychological disorders, mental disorders, and other systemic disorders.

### Isolation and culture of NP cells

NP cells from three patients in each group (L4/5; mean age, 55.16 years; 4 males and 8 females) were isolated as previously described [16]. After isolation, cells were maintained in Dulbecco’s modified Eagle’s medium supplemented with 10% fetal bovine serum and penicillin/streptomycin. For hypoxic culture, NP cells were cultured in a tri-gas incubator (Huaxi Electronics Technetronic Co., Ltd.) containing 1% O_2_, 5% CO_2_, and 94% N_2_ for 8 h.

### Protein isolation and Western blotting

NP tissues from three patients in each group were extracted and immediately placed on ice. The tissues were washed with pre-cooled PBS and homogenized using RIPA buffer (Aspen, China) supplemented with phenylmethanesulfonyl fluoride and protease and phosphatase inhibitors (Aspen, China). Tissue lysates were sonicated on ice, and protein concentrations were determined using a BCA protein assay kit (Sigma). Total protein was separated via SDS-PAGE on 10% gels and subsequently transferred onto PVDF membranes (EMD Millipore Corporation, USA) at 250 mA. After blocking with 5% non-fat dried milk in TBST at room temperature for 1 h, the membranes were incubated with anti-Notch1 (Abcam, ab44986), anti-NICD (Abcam, ab83232), anti-Notch2 (CST, 5732), anti-Notch3 (CST, 5276), anti-Notch4 (CST, 2423), anti-HIF-1α (Novus, NB100–105), anti-HES1 (Abcam, ab49170), and anti-GAPDH (ProteinTech, 60,004–1-Ig) primary antibodies overnight at 4 °C. After washing with TBST three times, the protein bands were incubated using HRP-conjugated secondary antibodies (ProteinTech) at room temperature for 1 h. The protein bands were detected using enhanced chemiluminescence detection reagents.

### RNA extraction and real-time polymerase chain reaction (RT-PCR)

Total RNA was isolated from NP tissues frozen in liquid nitrogen using the RNeasy Mini kit (Qiagen, Valencia, CA) according to the manufacturer’s instructions. The quantity of the total RNA extracted was determined using a spectrophotometer. Total RNA was reversed transcribed into complementary DNA using the First Strand cDNA Synthesis Kit (TAKARA, Japan) according to the manufacturer’s protocol. The forward and reverse primers used for quantitative PCR are presented in Table 2. Quantitative PCR was performed in triplicate in a 96-well plate using the KAPA SYBR FAST qPCR Kit Master Mix. Target gene mRNA levels were quantified using the 2^−ΔΔCt^ method and normalized to the internal reference gene GAPDH.

**Table 2:** Primer sequences used for the Real-Time Polymerase Chain Reaction

Gene		Primer sequence
Notch 1	Forward	F5’-GCGACAACGCCTACCTCTG
	Reverse	R5’-AAGCCATTGATGCCGTCC
Notch 2	Forward	F5’-TCAGCCGGGATACCTATGAG
	Reverse	R5’-CTGGCAGTGTCCTGGAATGT
Notch 3	Forward	F5’-ACCGCGTGGCTTCTTTCTA
	Reverse	R5’-GAGCACTCGTCCACATCCTG
Notch 4	Forward	F5’-GGAGAAGGGGCTGTGGAAT
	Reverse	R5’-GCAGGGGTCAGGAAACTGG
HES 1	Forward	F5’-GAGTGCATGAACGAGGTGAC
	Reverse	R5’-GGTCATGGCATTGATCTGG
HIF1α	Forward	F5’-ACCACCTATGACCTGCTTGG
	Reverse	R5’-TATCCAGGCTGTGTCGACTG
GAPDH	Forward	F5’-GCACCGTCAAGGCTGAGAAC
	Reverse	R5’-TGGTGAAGACGCCAGTGGA

### Immunohistochemical analysis

Paraffin-embedded NP tissue sections were deparaffinized in xylene and rehydrated through graded concentrations of ethanol. The sections were incubated with rabbit primary antibodies targeting HIF-1α and NICD overnight at 4 °C. Pre-immune rabbit nonspecific IgG antibody was used as a negative control. Immunopositive NP cells were counted in random fields of view and the results were expressed as a percentage of the total cells.

### Statistical analysis

All experiments were performed in triplicate. Data were analyzed using GraphPad Prism statistical software (version 7.00, La Jolla, CA, USA). The unpaired t-test or analysis of variance was used to compare the different groups, as applicable. Correlations between the MCs and facet joint or disc degeneration were evaluated using Spearman’s correlation analysis. *P* < 0.05 was considered to indicate a statistically significant difference.

## Results

### Histological analysis

**Fig. 2:**
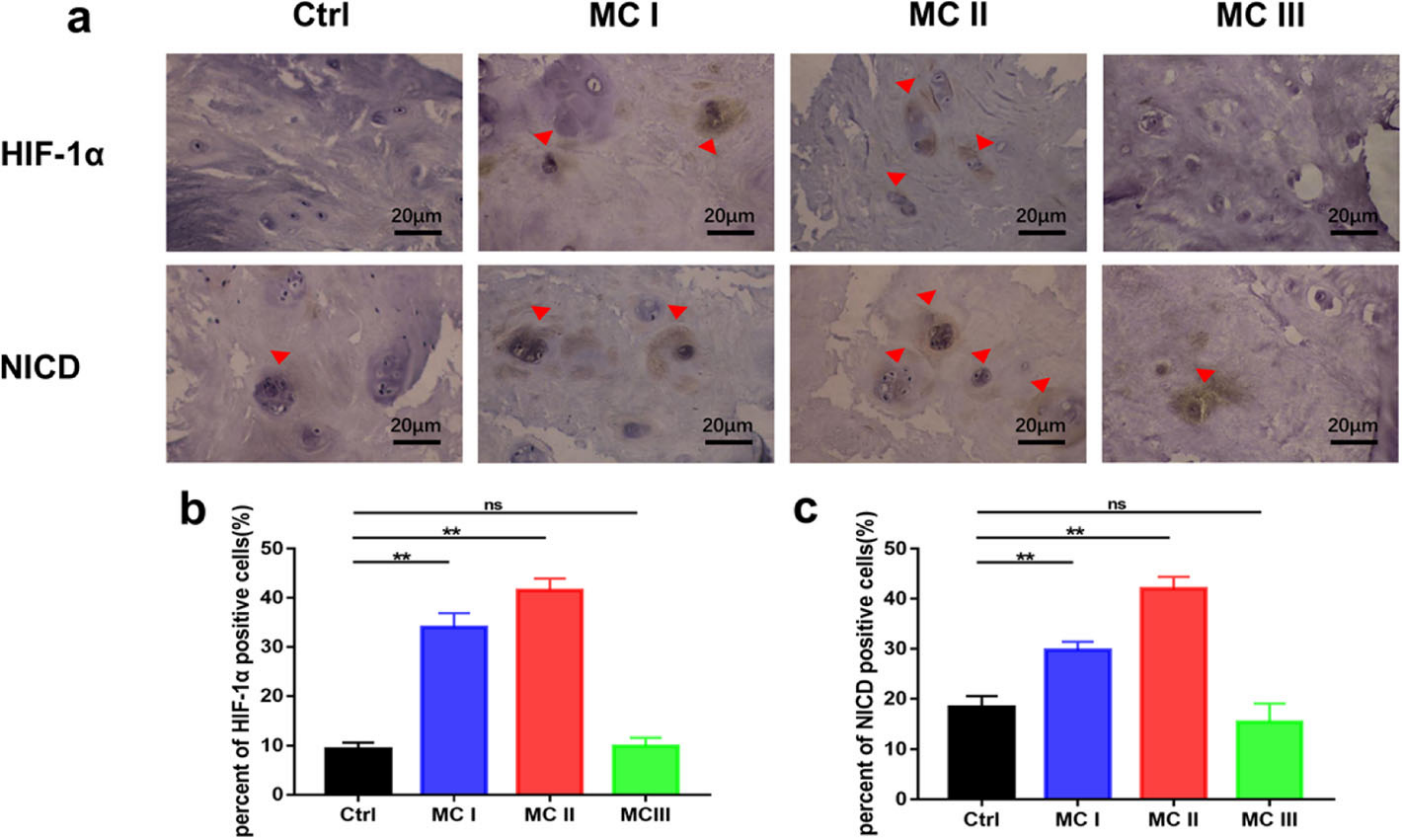
Immunohistochemistry of HIF-1α and the NICD in nucleus pulposus cells obtained from patients with different MCs and the control group. Scale bar: 20 μm. Values are presented as the mean ± S.E.M. *p < 0.05, **p < 0.01. NICD, Notch1 intracellular domain; MCs, Modic changes

HIF-1α expression was analyzed in human NP tissues isolated from degenerated IVDs. The results revealed that NP tissues obtained from patients in the MC I and MC II groups expressed higher levels of HIF-1α protein than did those from the control group. The expression of HIF-1α protein in the MC III group was not significantly different from that in the control group (Fig. 2a). The percentage of cells positive for HIF-1α in the MC I and MC II groups was significantly higher than that in the control group (MC I: 33.96 vs. 9.8%, MC II: 41.52 vs. 9.8%, *p* < 0.01; Fig. 2b). The expression of Notch1 intracellular domain (NICD) was correlated to the increased expression of HIF-1α in the MC I and MC II groups (MC I: 29.75 vs. 18.32%, MC II: 41.96 vs. 18.32%, *p* < 0.01; Fig. 2c).

### Gene expression

**Fig. 3:**
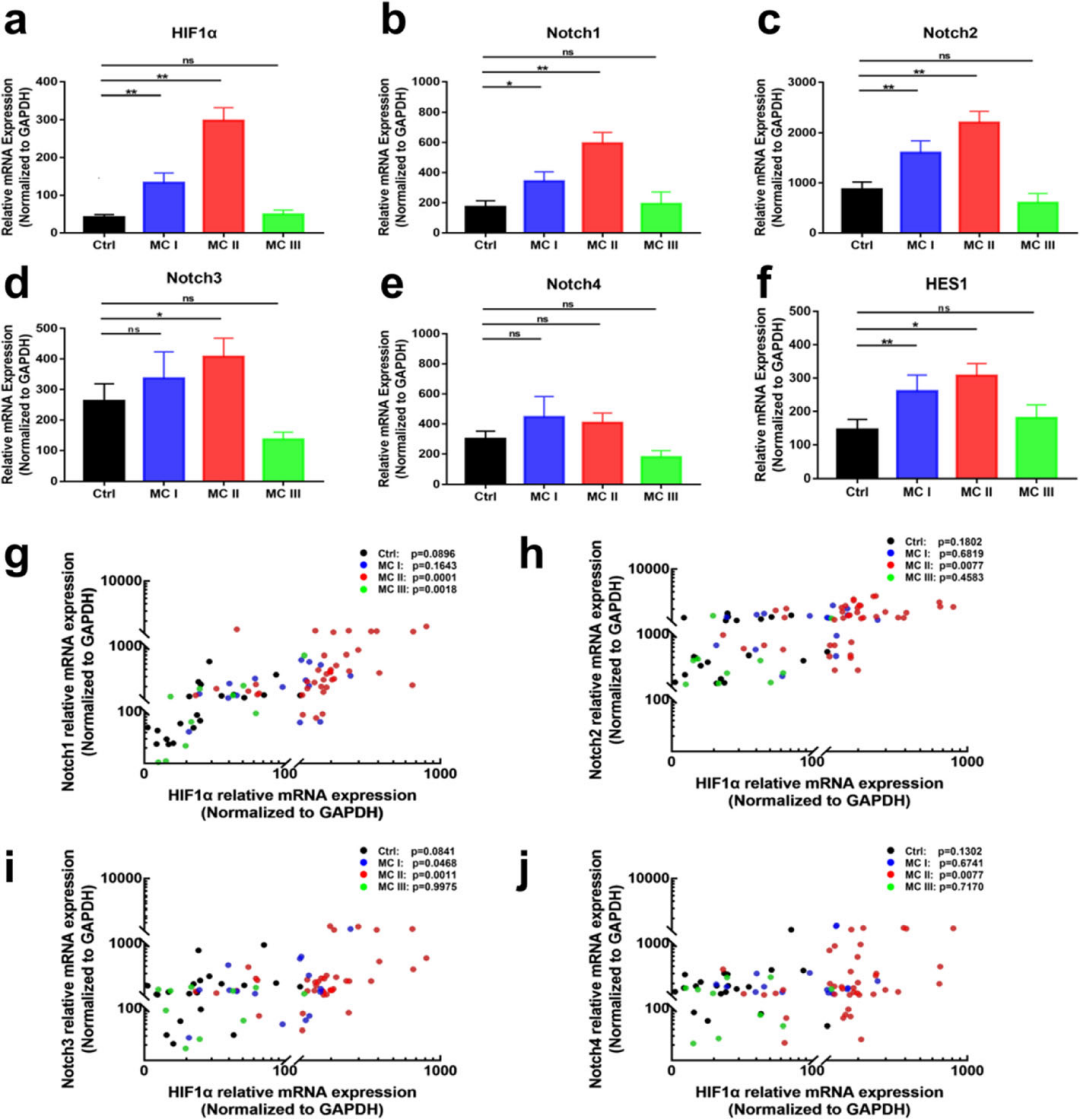
RT-PCR analysis of the mRNA levels of HIF-1α (**a**), Notch receptors (**b**-**e**), and HES1 (**f**) in tissues with different MCs. Spearman’s rank correlation analysis was performed to analyze the correlation between HIF-1α and Notch1 (**g**), HIF-1α and Notch2 (**h**), HIF-1α and Notch3 (**i**), and HIF-1α and Notch4 (**j**). Values are presented as the mean ± S.E.M. *p < 0.05, **p < 0.01

An increase in Notch1 and Notch2 mRNA levels was observed in the MC I and MC II groups, and this was positively correlated with upregulated HIF-1α expression. However, the expression of the aforementioned genes in patients with MC III was not statistically significantly different to that in the control group (Fig. 3a-c). Additionally, there was no significant difference in the expression of Notch3 in patients with MC I and MC III compared with that in the control group (Fig. 3d). The transcriptional level of Notch3 receptor in the MC II group was moderately increased compared with that in the control group. Levels of Notch4 transcripts in the MC groups were not significantly different to that in the control group (Fig. 3e). Moreover, the expression of HES1 (Fig. 3f), the target gene of Notch signaling, was significantly higher in the MC I and MC II groups than in the control group.

### Correlation analysis

The expression of HIF-1α and Notch1 (Fig. 3g; *p* = 0.0001), HIF-1α and Notch2 (Fig. 3h; *p* = 0.0077), and HIF-1α and Notch3 (Fig. 3i; *p* = 0.0011), and HIF-1α and Notch4 (Fig. 3j; *p* = 0.0077) in patients with MC II were significantly correlated with one another (Table 3). No significant correlations were observed between the LBP NRS and HIF-1α or Notch receptor expression in any of the 4 groups (Table 4).

**Table 3:** Correlation between the mRNA expression of HIF-1α and Notch receptors in different groups The *p* values and Pearson’s correlation coefficient (R^2^) are provided

	Ctrl		MC I		MC II		MC III	
	*p*	R^2^	*p*	R^2^	*p*	R^2^	*p*	R^2^
Notch1	0.09	0.15	0.16	0.14	**< 0.01**	**0.33**	**0.01**	**0.72**
Notch2	0.18	0.10	0.68	0.01	**0.01**	**0.17**	0.46	0.07
Notch3	0.08	0.16	**0.05**	**0.27**	**< 0.01**	**0.25**	1.00	< 0.01
Notch4	0.13	0.12	0.67	0.01	**0.01**	**0.17**	0.72	0.02

The *p* values and Pearson’s correlation coefficients (R^2^) are provided

**Table 4:** Correlation between the NRS scores and HIF-1α/Notch receptor mRNA expression in the different groups

	HIF-1α	Notch1	Notch2	Notch3	Notch4
MC I	0.66	0.20	0.99	0.84	0.89
MC II	0.67	0.54	0.83	0.92	0.12
MC III	0.63	0.79	0.74	0.84	0.77

*p* values are provided

### Protein expression in isolated NP cells

**Fig. 4:**
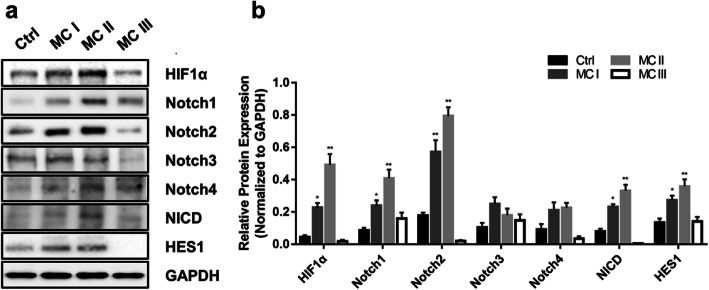
Representative protein expression of HIF-1α and Notch receptors/target genes in nucleus pulposus cells under hypoxic conditions (1% O_2_). GAPDH was used as the loading control. Values are expressed as the mean ± SEM of three individual experiments. *p < 0.05, **p < 0.01; (*n* = 3)

The isolated NP cells exhibited a HIF-1α-dependent increase in Notch1 and Notch2 protein levels (Fig. 4, *p* < 0.05). The expression levels of NICD and HES1 were significantly increased in the MC I and MC II groups compared with those in the control group, and this result was consistent with the trend observed for HIF-1α. These findings suggested that the expression of HIF-1α was correlated with the Notch signaling pathway in MC tissues.

## Discussion

The present study revealed that the expression of HIF-1α was elevated in NP cells obtained from patients with MC I and MC II compared with that in cells obtained from patients with pure disc herniation. HIF-1α is an indicator of anaerobic states. In response to hypoxic conditions, cells increase the synthesis of HIF proteins [17]. HIF-1α plays an important role in the regulation of the biological behaviors of NP cells [7, 10]. Therefore, the increased expression of HIF-1α is indicative of degeneration, ischemia or hypoxia. Furthermore, previous studies revealed that the HIF-1α/Notch signaling pathway plays an important role in anoxic pathological processes that occur in tumors and neurodegenerative diseases [18, 19]. Based on the aforementioned studies, we hypothesized that there is a correlation between Notch and hypoxia, through HIF-1α expression, in patients with IVD herniation. Our study demonstrated that the expression of NICD in NP cells obtained from patients with IVD protrusion was increased, and was positively correlated with HIF-1α levels. Therefore, we speculated that HIF-1α may affect Notch signaling and downstream molecules in the NP cells of patients with disc herniation.

Based on the immunohistochemical results obtained in the present study, we assessed the mRNA expression levels of major components of the Notch signaling pathway in lumbar disc cells obtained from patients with different MC types. There was an increased expression of Notch1, NICD, and HES1 in the MC I and MC II groups, which was consistent with the histological results obtained. The correlation between HIF-1α and the Notch signaling pathway suggests that HIF-1α regulates disc regeneration through activation of the Notch-HES1 pathway. Specifically, HIF-1α may promote recruitment of the NICD to the CSL-binding motifs in the HES1 promoter and maintain homeostasis of the extracellular matrix in the NP [20, 21].

Spearman’s rank correlation analysis was performed to assess the correlation between the NRS score and gene expression levels of HIF-1α and Notch receptors. Unlike the imaging methods used to evaluate the degree of IVD degeneration [22], the biochemical markers investigated in the present study were not positively correlated with the clinical symptoms of LBP in the different MC groups. This inconsistent result is likely because the Notch-HES1 pathway is not involved in the initiation of LBP. A previous study revealed that there were significant correlations between LBP and inflammatory factors such as IL-6, IL-8, PGE2, and TNFα [23, 24]. The majority of these factors have been successfully used to activate Notch signaling in NP [15, 25]. In addition, HIF-1α expression was significantly increased in IL-1β-stimulated NP cells under hypoxic conditions [26]. Moreover, extensive research shows that LBP may be the result of a low-grade infection caused by *Cutibacterium acnes* in MC I [27, 28]. The hypoxic microenvironment of IVD provides a favorable condition for the growth of anaerobic bacteria, thereby facilitating consistent accumulation of inflammatory cytokines (IL-8, MIP-1α, MCP-1, IP-10, TNF-α) [29]. We therefore speculated that upregulated inflammatory factors and low-grade bacterial infection participate in the activation of the Notch and HIF-1α pathways and subsequent initiation of IVD degeneration in patients with MCs, particularly MC I and MC II [30].

Several studies have demonstrated crosstalk between the HIF-1α and Notch signaling pathways in IVD. To the best of our knowledge, the present study was the first to elucidate the co-expression patterns of the HIF-1α and the Notch signaling pathway in patients with different MCs. Specifically, hypoxia-induced Notch receptors and downstream molecules were highly expressed in patients with MC I and MC II, but not MC III, as detected by RT-PCR, western blotting, and immunohistochemistry. Furthermore, Notch1 and Notch2 mRNA levels were markedly elevated in the NP, while Notch3 and Notch4 levels were not altered as a result of the change in oxygen concentrations in IVDs with MCs.

Collectively, the results of the present study revealed that the HIF-1α and Notch signaling pathways play an important role in IVD degeneration. Therefore, these pathways may serve as novel therapeutic targets, particularly for patients who are ineligible for surgery. Prior to future clinical application, further investigation of the interaction between HIF-1α and Notch signaling and the influence of downstream molecules is required.

This study had certain limitations. Firstly, the small sample size in the MC I and MC III groups could have led to a large error when conducting the Spearman’s rank correlation analysis, and may have influenced the statistical correlation between HIF-1α and Notch1/Notch2 in the MC I and MC III groups. Secondly, as the AF and endplate (EP) sections of the IVD samples were too small to allow follow-up analysis, they were carefully excluded from the NP tissue. Therefore, we did not evaluate changes in gene and protein expression in AF and endplate tissues, and further studies are required. Furthermore, the samples used for western blotting must exhibit strong proliferative ability in vitro. The samples from L4/5 in each group may lead to a large margin of selection bias.

## Supplementary information

**Additional file 1: Figure S1.** Western blot anlysis of samples from different MCs patients were treated with CoCl_2_ (100 μM) for 24 h, or cultured in hypoxia condition for 4, 8, or 12 h.

## Data Availability

Available upon request from the corresponding author.

## Abbreviations

IVD: Intervertebral disc
MCs: Modic changes
HIF-1α: Hypoxia-inducible factor-1α
NP: Nucleus pulposus
MRI: Magnetic resonance images
LBP: Low back pain
NICD: Notch1 intracellular domain
AF: Annulus fibrosus
