# Supplementary material for: Correlation between the HIF-1α/Notch signaling pathway and Modic changes in nucleus pulposus cells isolated from patients with low back pain
Source: BMC Musculoskelet Disord. 2020 Jul 28;21:500. doi: 10.1186/s12891-020-03505-w (PMC7390108; doi:10.1186/s12891-020-03505-w)
Supplement: Supplementary file 1 — Additional file 1: Figure S1. Western blot anlysis of samples from different MCs patients were treated with CoCl2 (100 μM) for 24 h, or cultured in hypoxia condition for 4, 8, or 12 h. [file 12891_2020_3505_MOESM1_ESM.docx]

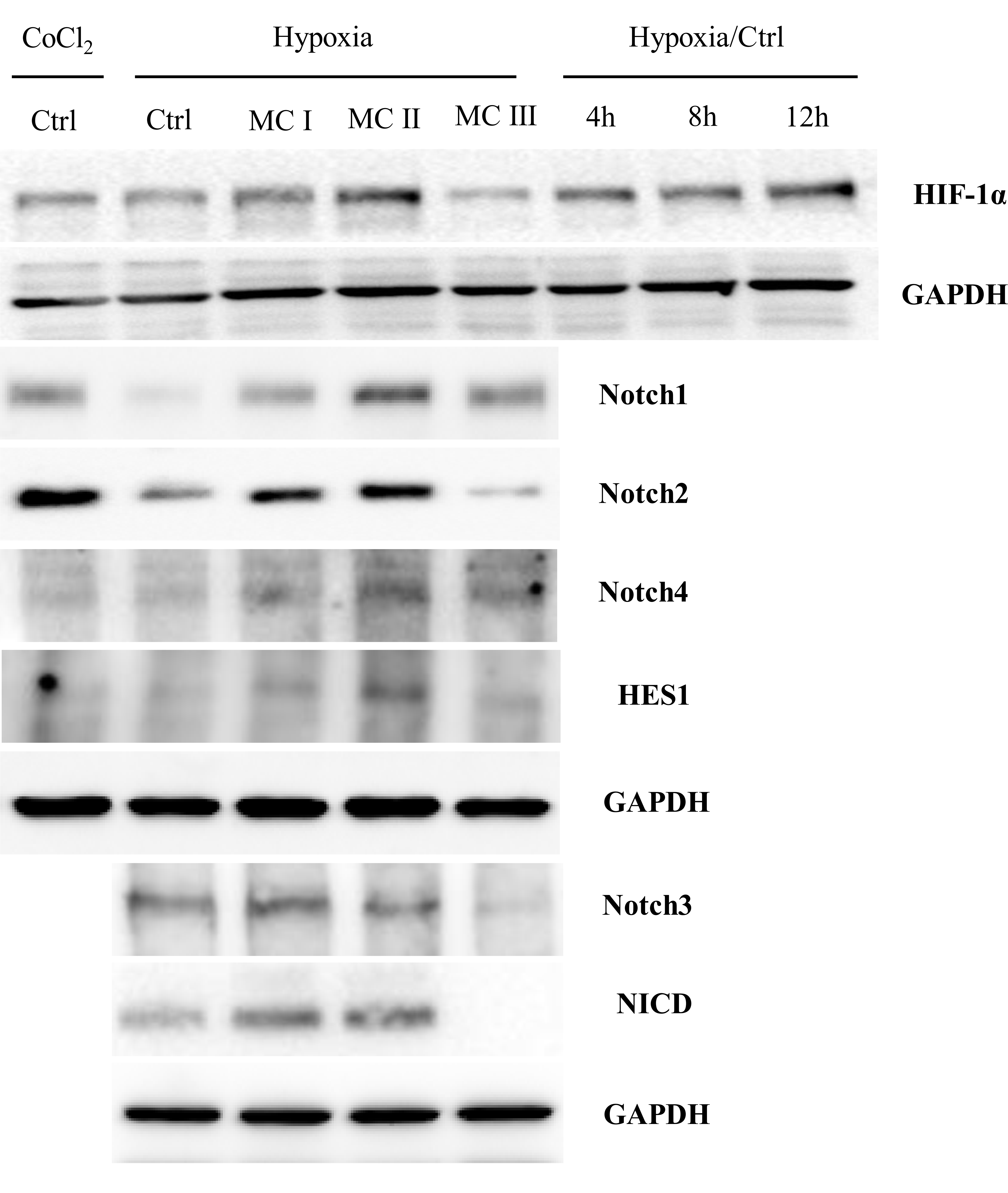


Fig S：Western blot anlysis of samples from different MCs patients were treated with CoCl_2_ (100μM) for 24h, or cultured in hypoxia condition for 4, 8, or 12h.
